# Risk Assessment and Implication of Human Exposure to Road Dust Heavy Metals in Jeddah, Saudi Arabia

**DOI:** 10.3390/ijerph15010036

**Published:** 2017-12-26

**Authors:** Ibrahim I. Shabbaj, Mansour A. Alghamdi, Magdy Shamy, Salwa K. Hassan, Musaab M. Alsharif, Mamdouh I. Khoder

**Affiliations:** 1 Department of Environmental Sciences, Faculty of Meteorology, Environment and Arid Land Agriculture, King Abdulaziz University, P.O. Box 80208, Jeddah 21589, Saudi Arabia; ishabbaj@kau.edu.sa (I.I.S.); mshamy@kau.edu.sa (M.S.); mkhader@kau.edu.sa (M.I.K.); 2 Air Pollution Department, National Research Centre, El Behooth Str., 12622 Dokki, Giza , Egypt; salwakamal1999@gmail.com; 3 Faculty of Medicine, King Abdulaziz University, Jeddah 21589, Saudi Arabia; malsharif0069@stu.kau.edu.sa

**Keywords:** urban road dust, functional areas, heavy metals, pollution assessment, health risk assessment, Jeddah

## Abstract

Data dealing with the assessment of heavy metal pollution in road dusts in Jeddah, Saudi Arabia and its implication to human health risk of human exposure to heavy metals, are scarce. Road dusts were collected from five different functional areas (traffic areas (TA), parking areas (PA), residential areas (RA), mixed residential commercial areas (MCRA) and suburban areas (SA)) in Jeddah and one in a rural area (RUA) in Hada Al Sham. We aimed to measure the pollution levels of heavy metals and estimate their health risk of human exposure applying risk assessment models described by United States Environmental Protection Agency (USEPA). Using geo-accumulation index (I_geo_), the pollution level of heavy metals in urban road dusts was in the following order Cd > As > Pb > Zn > Cu > Ni > Cr > V > Mn > Co > Fe. Urban road dust was found to be moderately to heavily contaminated with As, Pb and Zn, and heavily to extremely contaminated with Cd. Calculation of enrichment factor (EF) revealed that heavy metals in TA had the highest values compared to that of the other functional areas. Cd, As, Pb, Zn and Cu were severely enriched, while Mn, V, Co, Ni and Cr were moderately enriched. Fe was considered as a natural element and consequently excluded. The concentrations of heavy metals in road dusts of functional areas were in the following order: TA > PA > MCRA > SA > RA > RUA. The study revealed that both children and adults in all studied areas having health quotient (HQ) < 1 are at negligible non-carcinogenic risk. The only exception was for children exposed to As in TA. They had an ingestion health quotient (HQ_ing_) 1.18 and a health index (HI) 1.19. The most prominent exposure route was ingestion. The cancer risk for children and adults from exposure to Pb, Cd, Co, Ni, and Cr was found to be negligible (≤1 × 10^−6^).

## 1. Introduction

Due to rapid urbanization, population growth and increasing demand of land for development, urban areas are experiencing rapid change throughout the world including dramatic growth in both industrial and road traffic activity which places great pressure on the local environment [1,2]. Road dust, the accumulated particle on the ground road surfaces, is a heterogeneous mixture of different contaminants originating from natural and anthropogenic sources and from the interaction of solid, liquid and gaseous pollutants derived from different sources [3,4,5]. Road dust is related to particulate content in the atmosphere through re-suspension into and re-deposition from the atmosphere and is chemically similar, in some respects, to the primary portion of atmospheric particulate [6,7]. Therefore, road dust is a valuable medium for characterizing urban environmental quality [8] and its chemical composition is an indicator for environmental pollution [9].

Road dust is a main reservoir of metals in urban environment from surrounding areas [8,10]. Metals in road dust result from traffic emissions (exhausts, oil lubricants, vehicle wear, brake lining, corroding building-material asphalts, automobile parts and yellow road paint degradation), industrial emissions (smelters, incinerators, foundries and steel plants), as well as dry and wet deposition of atmospheric particulates [3,11,12]. In urban areas, traffic-related metal pollution in road dust is affected by vehicle type, traffic volume and behavior, soil parameters and meteorological conditions [3,12,13]. Recently, several studies investigated the contents, spatial distribution, source identification, contamination assessment and characterization of potentially toxic metals in road dust [8,14,15,16].

Metals enriched in the accumulated dust due to the lack of bioavailability, biodegradability and persistence pose a great deal of risk to human health through direct and indirect human exposure [17]. Ingestion and inhalation are the direct exposure pathways, while dermal contact and outfits are the indirect ones [18,19]. Oral ingestion was identified as the most critical exposure route to street dust particles for humans, compared with dermal contact and inhalation [20,21,22,23,24]. Oral ingestion takes place inadvertently, with food and drink or via mucociliary clearance, and with respect to children, deliberately, through their hand to mouth activities [19,25,26]. However, only the oral bio-accessible fraction of heavy metals that is soluble in the gastrointestinal tract available for absorption represents the actual health risks in ingested particles [27,28].

Accumulation of heavy metals in the human body increases with exposure to high levels and affects the central nervous system, circulatory system, the functioning of internal organs, and the malfunction of endocrine system [10,18,21,29] and acts as a secondary factor for other diseases such as growth retardation in children, kidney disease and cancer [18,30,31,32,33]. According to the calculated hazard indices, exposure to Hg, Pb, Zn, Cd and Mn in road dust was found to pose high potential ecological risk [2,34].

Recently, metal contamination of the road dusts has received much attention to assess the quality of the environment, identify pollution sources and investigate their adverse health effects [2,34,35,36,37,38]. Most of the previous studies on heavy metals pollution in Jeddah focused on their concentrations in street dust [39,40] and their levels, sources and health risk in suspended particulate matter [41,42,43,44]. However, data concerning evaluation, spatial distribution and health risk of heavy metals in road dust in different functional areas in Jeddah are scarce. Therefore, the main objectives of the current study were as follows: (1) to assess the pollution level and compare the concentrations and spatial patterns of heavy metals in road dusts in different functional areas of Jeddah; and (2) to investigate carcinogenic and non-carcinogenic health risks due to heavy metals exposure in children and adults.

## 2. Materials and Methods

### 2.1. Study Area

With increasing developmental activity, environmental concerns are increasing in Saudi Arabia [45]. Jeddah lies on the Red Sea coast in the western part of Saudi Arabia and is surrounded by mountains from north-eastern, eastern and south-eastern sides (latitude 29.2 North and longitude 39.7 East). It is the largest city in Saudi Arabia, with a land area of 1765 km^2^, and represents a very important commercial center, in addition of being the crossroads between East and West to Asia, Africa and Europe, with a population of ca. 3.6 million. Jeddah receives approximately 2 million visitors during pilgrimage season each year. Road traffic and stationary sources are the main sources emission of air pollutants in Jeddah. Jeddah experiences a huge traffic congestion due to increasing population and growing number of commuters. More than 1.40 million vehicles/day are running in the streets of Jeddah city [41]. These vehicles use mainly unleaded gasoline and diesel fuels. Oil refinery, seaport activities, desalination plant, power-generation plant and industrial activities in the south are the main stationary sources in the city. Jeddah has an arid climate, warm and humid or moderate in winter, and is characterized by high temperature, humidity, and solar radiation in summer. Rainfall is generally sparse.

### 2.2. Sampling Collection

**Figure 1:**
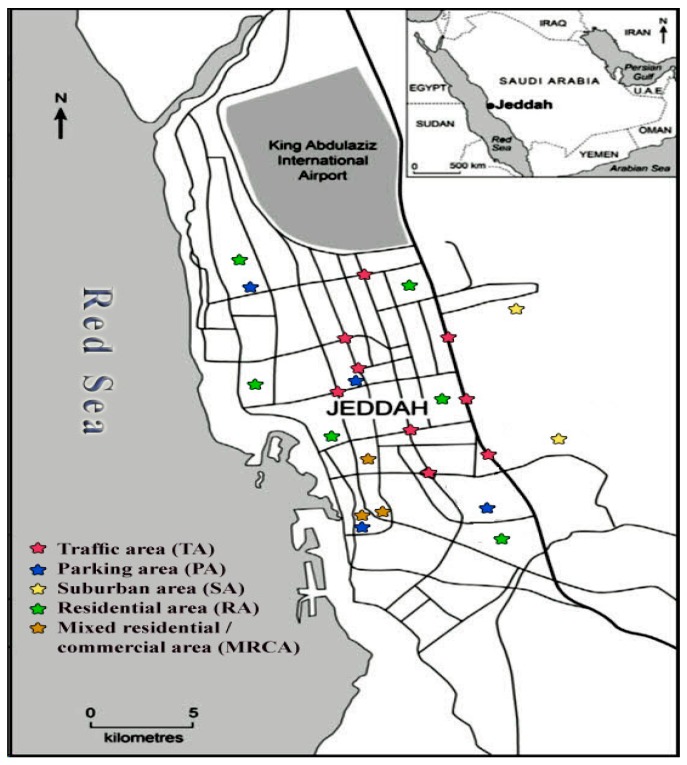
Sampling site distribution in the different functional areas of Jeddah.

Road dusts samples were collected from five different functional areas in Jeddah and one rural area in Hada Al Sham. The sampling locations (Figure 1) were distributed over the areas that represent various functional categories to reveal the pollution impacts from various human activities; including residential areas (RA), suburban areas (SA), mixed commercial/residential areas (MCRA), parking areas (PA), traffic areas (TR), and one rural area (RUA). The traffic areas included in this study cover a major highway, roundabouts and crossroads. The RUA is located at Hada Al Sham, about 60 km east of the city of Jeddah. Road dust samples were collected on the driest month of the year (September 2016). Samples at each sampling location (approximately 200 g each) were collected by gentle sweeping motion of an appropriate area from the pavement on both sides of the roads using a soft polyethylene brush and dustpan, thus ensuring that the samples were collected from the surface soil. The collected samples were stored in labeled sealed polypropylene bags and transported to the lab.

### 2.3. Sample Preparation and Analysis

In the laboratory, the samples were air-dried at room temperature and the coarse impurities of the samples, large plant/animal/biological parts, as well as, irrelevant gravel-sized materials, were removed using 1.0 mm mesh nylon. The rest was homogenized and sieved through 63 μm sieve size and stored in small self-sealing plastic bags for analysis. Only, dust with particle size <63 µm diameter was selected to determine its metal concentration in this study because: (1) metal concentration decreases with increase particle size of dust [46,47], (2) they represent high health risks [8,24] and (3) are easily transported and remain airborne for considerable durations [2,35,48].

### 2.4. Sample Digestion and Analysis

To measure heavy metal concentration, accurately weighed road dust samples (1 g) were digested with nitric acid (HNO_3_) and hydrochloric acid (HCl) mixture on a hot plate as described by Hassan and Khoder [49]. The digested solutions were filtered through Whatman filter paper (No. 42) using deionized water and diluted to 100 mL. They were stored at 4 °C in pre-cleaned polyethylene bottles until analysis. Inductively Coupled Plasma Optical Emission Spectrometry ICP-OES-5100 was used to determine the concentrations of heavy metals (Fe, Mn, Zn, Pb, Cd, V, Co, Ni, Cr and Cu) and As. The quality of data was ensured using standard material between samples. For quality assurance/quality control (QA/QC) and precision of measured metals, laboratory blanks, filter blanks and reagent blanks and certified soil reference material (soil CRM: NIST 2710) were analyzed. Mean recoveries for the studied elements (C (element, measured)/C (element, certified) × 100) in the CRM were between 73.2% and 102.9% (Supplementary information: Table S1). The precision of measured metals, determined from the standard deviation of repeated measurements of standards, was less than 2.5%. The concentration of metals in laboratory blanks, filter blanks and reagent blanks were measured by the same method described above in order to evaluate external metal contamination from analytical procedures. No contamination was detected.

### 2.5. Pollution Assessment Methodology

#### 2.5.1. Geo-Accumulation Index (I_geo_)

The geo-accumulation index (I_geo_) was used to evaluate the contamination levels of metal in road dust [50]. This index is widely applied to assess the heavy metal pollution of urban road dusts [17,51]. It assesses the metal pollution in terms of seven enrichment classes ranging from (0–6), starting from “normal background value” to “very heavily polluted” [17,50]. The seven different classes for I_geo_ values are given in Table 1. The I_geo_ was computed from the following equation [52]

(1) $$I_{\mathrm{geo}}=\log_{2}\left(C_{n}/1.5B_{n}\right)$$

where I_geo_ is the geo-accumulation index for different metals and C_n_ the measured concentration of the metals in road dust samples. The constant 1.5 is used to minimize the effect of possible variations in the background values. B_n_ refers to the metal background value in the earth’s crust [53].

#### 2.5.2. Enrichment Factor

The enrichment factor (EF) was used to differentiate between the anthropogenic sources of trace metals and their natural origin in road dust, as well as, to evaluate the degree of the anthropogenic contribution and metal contamination. It was calculated using the following equation [55,56].

(2) $$\mathrm{EF}=\frac{{\left(C_{x}/C_{\mathrm{reference}}\right)}_{\mathrm{Road}\;\mathrm{dust}}}{{\left(C_{x}/C_{\mathrm{reference}}\right)}_{\mathrm{Earth}\;\mathrm{crust}}}$$

where EF is the enrichment factor, C_x_ the concentration of the target metal, and C_Reference_ the concentration of the reference metal. In the present study, Fe was chosen as a reference metal and was used for EF calculation. The earth crust composition was taken from Taylor [53] and Taylor and McLennan [57]. Using average crust values provides a meaningful comparison to many other studies that commonly use this technique. The average local soil profiles are not available. An EF values <2 indicate deficiency to minimal enrichment [58]. EF values between 2 and 10 refer to moderate enrichment, whereas EF values >10 show severe enrichment [59].

### 2.6. Health Risk Assessment Model

Health risk assessment models were used to quantify the health risk (carcinogenic and non-carcinogenic) for children and adults exposed to heavy metals in road dust. They are based on those developed by the United States Environmental Protection Agency (USEPA) [60,61]. Local residents are exposed to metals in road dust through three main exposure pathways: direct ingestion, inhalation through mouth and nose, and dermal absorption. The total non-carcinogenic risk was calculated for each metal in road dust by the summation of the individual risks calculated for the three exposure pathways [60,62].

The average daily dose (ADD) (mg kg^−1^ day^−1^) for heavy metals in road dust through the three exposure pathways was calculated according to Exposure Factors Handbook [63] and the Technical Report of USEPA [64] using the following equations

(3) $${\mathrm{ADD}}_{\mathrm{ing}}=\frac{C\times \mathrm{IngR}\times \mathrm{CF}\times \mathrm{EF}\times \mathrm{ED}}{\mathrm{BW}\times \mathrm{AT}}$$

(4) $${\mathrm{ADD}}_{\mathrm{inh}}=\frac{C\times \mathrm{InhR}\times \mathrm{EF}\times \mathrm{ED}}{\mathrm{PEF}\times \mathrm{BW}\times \mathrm{AT}}$$

(5) $${\mathrm{ADD}}_{\mathrm{dermal}}=\frac{C\times \mathrm{SA}\times \mathrm{CF}\times \mathrm{AF}\times \mathrm{ABF}\times \mathrm{EF}\times \mathrm{ED}}{\mathrm{BW}\times \mathrm{AT}}$$

(6) $$\mathrm{LADD}=\frac{C\times \mathrm{CR}\times \mathrm{EF}\times \mathrm{ED}}{\mathrm{PEF}\times \mathrm{BW}\times \mathrm{AT}}$$

where the ADD_ing_, ADD_inh_ and ADD_dermal_ are the average daily dose (mg kg^−1^ day^−1^) exposure to metals through ingestion, inhalation and dermal contact, respectively. LADD is the lifetime average daily dose exposure to metals (mg kg^−1^ day^−1^) for cancer risk, CR is the contact frequency and is the same IngR used in the calculation of ADD_ing_ [64,65,66]. The detailed description of the values of exposure factors for children and adults applied to the above models (Equations (3)–(6)) are given in Table 2.

In order to evaluate the human health risk of heavy metal exposure from road dusts in Jeddah, the HQ (hazard quotient), HI (hazards index), and CRA (carcinogenic risk assessment) were applied. The potential risk of carcinogenic and non-carcinogenic hazards for individual metals were calculated using the following equations [72,78]:

(7) $$\mathrm{HQ}=\frac{\mathrm{ADD}}{\mathrm{RfD}}$$

(8) $$\mathrm{HI}=\sum {\mathrm{HQ}}_{i}$$

(9) CRA=LADD×SF

where RfD and SF are the values of reference dose (mg kg^−1^ day^−1^) and slope factor [36,65,66,79,80]. RfD is an estimation of maximum permissible risks to human population through daily exposure by considering sensitive group (children) during a lifetime [17].

The carcinogenic risk is the probability of an individual developing any type of cancer from lifetime exposure to carcinogenic hazards [18,21]. It is recommended that the value of CRA < 1 × 10^−6^ can be regarded as negligible, whereas CRA > 1 × 10^−4^ is likely to be harmful to human beings. The acceptable or tolerable risk for regulatory purposes is in the range of 1 × 10^−6^ ~ 1 × 10^−4^ [60,65,66]. There are no adverse health effects when the value of HQ ≤ 1, whereas adverse health effects occur when HQ > 1 [60]. HI value show the sum of the value of the HQ for different substance through different pathways [18,81] and refers to total risk of non-carcinogenic for a single metal. The value of HI ≤ 1 refers that no significant risk of non-carcinogenic effects is occur. On the other hand, there is a chance that non-carcinogenic effects may occur when HI >1, and the probability increase with increasing the value of HI [65,66].

## 3. Results and Discussion

### 3.1. Heavy Metals Concentration in Urban Road Dusts

**Figure 2:**
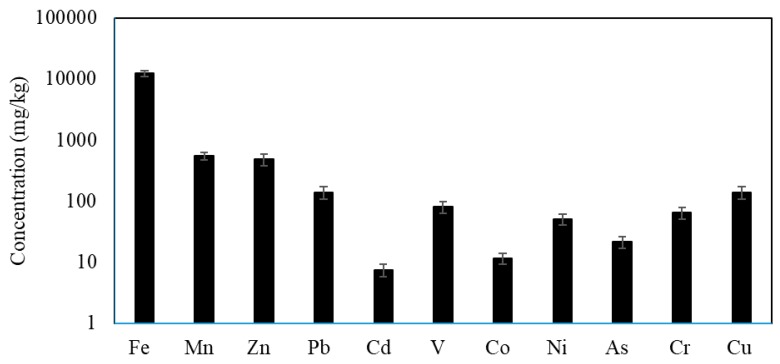
Average heavy metals concentrations in the urban road dusts of Jeddah.

The average concentrations of heavy metals in urban road dusts collected from Jeddah are shown in Figure 2. The mean concentrations of heavy metal in descending order were Fe > Mn > Zn > Pb > Cu > V > Cr > Ni > As > Co. and Cd. The mean concentrations were 12,449.45, 550.61, 487.52, 140.73, 7.46, 80.92, 11.66, 51.29, 21.55, 65.43 and 139.11 mg/kg for Fe, Mn, Zn, Pb, Cd, V, Co, Ni, As, Cr and Cu, respectively. The concentrations of heavy metals in urban road dusts exceeded the rural values except for Fe and Mn. Their mean values were 6.02, 9.25, 18.65, 2.32, 2.53, 2.33, 9.58, 1.59 and 6.89 fold higher than those in the RUA for Zn, Pb, Cd, V, Co., Ni, As, Cr and Cu, respectively, indicating that the metal pollution in urban road dusts might derive mainly from anthropogenic sources [8,14,17]. Fe concentrations were lower in urban than rural dusts, while Mn concentrations in both urban and rural dusts were nearly similar. The maximum permissible concentrations (MPC) for Pb, Cu, Mn, Zn, Co. and Cd in soil are 100, 100, 1500, 300, 30, and 3 mg/kg, respectively [82]. In the present study, only the concentration of Zn, Pb, Cd and Cu were higher than the MPC.

The spatial variations of heavy metals concentrations in road dusts from different functional areas are shown in Table 3. The concentrations of all heavy metals (except Fe in all sites and Mn in RA and SA) in RA, SA, MCRA, PA and TA dusts were higher than rural values, assuming that the heavy metals in urban road dusts might be contaminated by anthropogenic activities like vehicular traffic, building construction and demolition activities and waste disposal [36]. Fe concentration in urban dusts was lower than that in RUA, supporting that it mostly comes from natural sources. Based on the total heavy metals (Mn, Zn, Pb, Cd, V, Co, Ni, As, Cr and Cu) concentrations, functional areas in Jeddah could be classified as follows: TA > PA > MCRA > SA > RA. The observed high concentrations of the total heavy metals in the road dusts of TA suggest that the TA areas may be a reservoir of heavy metals in this urban environment. Traffic area (TA) covers a major highway, roundabouts and crossroads with the highest traffic volumes and traffic jams in Jeddah. Therefore, the vehicular- related deposition of particles might be responsible for higher concentrations of metals in road dusts of TA. Previous studies reported that deposited particles come from vehicle exhaust, lubricating oil residues, tire wear, brake lining wear, atmospheric deposition, plant matter, and materials produced by the erosion of the adjacent soil [74,83,84,85,86]. Generally, the urban area is an assembly of different land use types with typical local and diffuse pollution sources. So, the wide variations in heavy metals concentrations between different functional areas might be attributed to the distinctive artificial activities in each functional area that release different kinds of heavy metals which are deposited in the street surface [87,88].

Comparison of heavy metals concentrations in road dust of Jeddah with those in other cities in the world is shown in Table 4. In general, the concentrations of heavy metals in Jeddah road dust were lower/higher or similar to those reported in other cities. These variations might be referred to the difference in the traffic density, intensity of human activities, land use patterns, technologies employed, and local weather conditions [2]. For example, the mean concentration of Cu in Jeddah road dust is almost similar to Iran (Shuraz) and UK, lower than Colombia, Iran (Tahran, Asfhan), Jordan and China (Guangzhou), and higher than China (Chengdu, Beijing, Baoji, Nanjing, Xian), USA, Turkey and Greece. Pb content in Jeddah road dust and Turkey are similar or higher than USA, Iran (Shuraz) and China (Chengdu, Beijing and Nanjing), but lower than Colombia, Iran (Tahran and Isfahan), Greece, Jordan, and China (Guanghou, Baoji and Xian). On the other hand, As and Cd levels in road dust of Jeddah were higher than all fore-mentioned cities. These results support the idea that each city has its own characteristic combination of metal composition, and the observed variations and similarities in heavy metal concentrations among the cities may not reflect the actual natural and anthropogenic diversities among different urban settings.

### 3.2. Assessment Urban Road Dusts Quality

The I_geo_ values for heavy metals in road dust from different functional areas are presented in Table 5. Road dusts in different areas have different I_geo_ values among the urban areas of Jeddah. The rank order for I_geo_ values in road dusts from RA, SA, MCRA, PA and TA were nearly similar, with highest values for Cd, As, Zn and Cu and lowest values for Fe and Co. The order for the average I_geo_ values in urban road dusts were Cd > As > Pb > Zn > Cu > Ni > Cr > V > Mn > Co > Fe. The I_geo_ values were <0 for Ni, Cr, V, Mn, Co and Fe, <1 for Cu and >1 for Cd, As, Pb and Zn (Table 5). According to the criteria of contamination of urban road dusts based on I_geo_ (Table 1), urban road dusts of Jeddah was uncontaminated by Ni, Cr, V, Mn, Co and Fe; uncontaminated to moderately contaminated by Cu; moderately to heavily contaminated by As, Pb and Zn; and heavily to extremely contaminated by Cd. Increased socio-economic activities in urban areas and lack of proper disposal protocols of products like paint, oil, greases, fuel and used tires might have increased metal contamination [54,89]. Shi et al. [62] and Garcia-Martinez and Poleto [90] reported high average I_geo_ value of Pb in urban areas. The highest level of pollution was found for As having I_geo_ values of more than six in the metropolitan area of Hefei, China [36].

The EF for each heavy metal in road dusts from different functional areas are shown in Table 5. EF values lower than 2 were found for V and Co in RA, Co in SA and Mn, Cr, Cu, V, Co and Ni in RUA, indicating that these metals originate from natural sources such as crustal erosion and wind- blown soil minerals. The EF values of Mn, Ni, Cr, and Cu in RA, Mn, V, Ni, Cr, and Cu in SA, Mn, V, Co, Ni, and Cr in MCRA, Mn, V, Co., Ni, and Cr in PA, Mn, V, Co, Ni, and Cr in TA and Zn, Pb and As in RUA were between 2 and 10. Furthermore, the EF values for Zn, Pb, Cd and As in RA and SA, Zn, Pb, Cd, As and Cu in MCRA, PA and TA were more than 10. Generally, the mean EF values in the urban road dusts of Jeddah displayed the following decreasing trend: Cd > As > Pb > Zn > Cu > Ni > Cr > V > Mn >Co. The mean EF values of Mn, V, Co, Ni and Cr were between 2 and 10, indicating that they were moderately enriched. For Cd, As, Pb, Zn and Cu, they were more than 10, indicating that they were severely enriched. Cu, Pb and Zn are reported to be multi-source related and their accumulation is commonly found to be anthropogenic and from traffic related materials (brake dust, tires tread and yellow paint) [96]. Moreover, high atmospheric temperature and exposure to weather may accelerate corrosion processes, causing wear of the wares, walls, lamps and railings that often contain the heavy metals such as Zn, Cu, Cd and Cr, resulting in the release of the metals to the urban environment and their accumulation in urban street dust [14,87,97]. Although the legal usage of leaded gasoline was phased out in Saudi Arabia in 2001 [98], the observed elevated Pb in urban road dusts of Jeddah may be attributed to historical Pb contamination and the long half-life of Pb in soils [99].

### 3.3. Human Health Risk Assessment

Human health risk assessment of heavy metals in the road dusts of different functional areas through possible exposure pathways (ingestion, inhalation, and dermal contact) was performed for children and adults (Table 6 and Table 7). Based on the calculated HQ values for the ingestion (HQ_ing_) and dermal (HQ_dermal_) pathways for children and adults exposed to heavy metals in road dusts, the rank order of functional areas was TA > PA > MCRA > SA > RA > RUA. While the rank order for inhalation (HQ_inh_) pathway was RUA > TA > SA = RA > PA > MCRA.

Concerning the heavy metals, As, Pb and Cr displayed higher HQ_ing_, Fe, Mn and Cr displayed higher HQ_inh_ and finally Cr, V and Cd displayed higher HO_demal_ for both children and adults compared with the other elements in the studied areas. An exception was found in RUA, where Cr, Mn and As showed higher HQ_ing_, whereas Cr, V and Mn showed higher HQ_dermal._

Results revealed that no non-carcinogenic significant risk was found in the study areas for all measured heavy metals, since the HQs and HI values were <1 [61]. The only exception was for HQ_ing_ and HI values for children exposed to As in TA (1.18 and 1.19, respectively).

When the mean HQs of the five urban areas was calculated (Table 8), the average hazard quotient values of heavy metals for children and adults were in the order of As > Pb> Cr > Mn >V > Cd > Cu > Ni > Zn >Fe > Co. for HQ_ing_, Fe > Mn > Cr > Co. >As > Pb > V > Cd > Cu > Ni > Zn for HQ_inh_ and Cr > V > Cd > Mn > Pb > Fe > As > Cu > Ni > Zn > Co. for HQ_dermal_. The HQ values for the different exposure pathways of measured heavy metals in children and adults decreased in the following order: ingestion > dermal contact > inhalation. The contributions of the HQ_ing_, HQ_inh_ and HQ_dermal_ to the HI (the total risk of non-cacinogenic exposure) were 94.86%, 1.52% and 3.62% for children and 56.13%, 3.01% and 40.86% for adults, respectively. This indicates that ingestion was the main pathway exposure to the measured heavy metals in urban road dusts of Jeddah city in the two population groups. These results are consistent with those reported in other studies [4,8,17,18,19,35,36,62,100,101].

The cancer risk (CRA) for some selected heavy metals (Pb, Cd, Co, Ni, As and Cr) was estimated using inhalation mode of exposure. The CRA values for children and adults exposed to these heavy metals in road dusts from different functional areas are presented in Table 8 and Table 9. All CRA values for both populations were equal to or lower than 1 × 10^−6^, with higher values in children, suggesting that the carcinogenic risk from exposure to these metals is negligible. These results are similar to those reported in literature [8,34,36,102].

Special attention should be paid for children exposure. HQ_ing_ and HQ_inh_ values for children was higher than adults. The HQ for children through ingestion and inhalation was in average 9.33 and 2.79 times higher than that for adults, indicating that children face more potential harmful health risk through both ingestion and inhalation of heavy metals in road dusts from Jeddah city. Children are more vulnerable to dust exposure because of their playing habits (ingestion of dust through mouth, hand licking, toys and other household objects) [19,103].

It is noteworthy to insist here that, the computed HQ and CRA values might not be low enough to allow for additional exposures and thus may not be sufficiently protective of human health [104,105]. The present study deduced that there is no serious risk from heavy metals in road dusts via different exposure routes. However, the possibility that these metals can cause serious health effects by their accumulation in body tissues persists [103,106,107]. Moreover, exposure to heavy metals through dust is only one of the major human exposure pathways of the contaminants, other routes of exposure must be considered. In Saudi Arabia, drinking water, especially water wells [108], vegetables [109], fruits [110], cereals [111], spices and herbs [112], cow’s milk [113] and fishes [114,115] were reported to be contaminated with heavy metals in different levels. Therefore, human risk assessment studies based on all possible exposures are highly recommended in Saudi Arabia.

## 4. Conclusions

This study aimed to find out the concentration, spatial variation, pollution level and health risk implication of human exposure to heavy metals (Fe, Mn, Zn, Pb, Cd, V, Co., Ni, Cr and Cu) and As in road dusts from five different functional areas in Jeddah and one rural area at Hada Al Sham, located about 60 km from the city of Jeddah. The average concentrations of Zn, Pb, Cd, V, Co, Ni, As, Cr and Cu in urban road dusts were higher than in rural area, indicating that this pollution may result from anthropogenic sources. Among the five urban areas, the highest levels of Mn, Zn, Pb, Cd, V, Co, Ni, As, Cr and Cu were found in TA and the lowest in RA. Based on Geo-accumulation Index (I_geo_), urban road dusts of Jeddah was uncontaminated by Ni, Cr, V, Mn, Co. and Fe, uncontaminated to moderately contaminated by Cu, moderately to heavily contaminated by As, Pb and Zn, and heavily to extremely contaminated by Cd. The order for the average I_geo_ values was Cd > As > Pb > Zn > Cu > Ni > Cr > V > Mn > Co > Fe in urban street dusts. The mean EF values in the urban road dusts of Jeddah displaying the following decreasing trend: Cd > As > Pb > Zn > Cu > Ni > Cr > V > Mn > Co. Cd, As, Pb, Zn and Cu in road dusts were severe enriched, whereas Mn, V, Co, Ni and Cr were moderately enriched. EF values of heavy metals in urban dusts were higher in TA than other functional areas. The HQs and HI values for the different exposure pathways of measured heavy metals in children and adults decreased in the following order: ingestion > dermal contact > inhalation. These values for all heavy metals in all functional areas were below the safe level (<1) indicating that no significant potential health risk is posed to inhabitants (children and adults) from exposure to heavy metals in road dusts, except for As with HQ*_ing_* value of 1.18 and HI value of 1.19 for children in TA. The HQ_ing_, HQ_inh_ and HI values were higher in children than adults. The carcinogenic risk (CRA) for heavy metals in Jeddah was found to be within the safe limits for children and adults, suggesting no potential harm from exposure to these metals in road dusts. Again, CRA values of heavy metals were higher in children than adults.

## Figures and Tables

**Table 1:** Value, classes and qualitative description of geo-accumulation index (I_geo_) *.

I_geo_ Value (log_2_ (x))	I_geo_ Class	Qualitative Designation of Road Dust
I_geo_ ≤ 0	0	Uncontaminated
0 < I_geo_ ≤ 1	1	Uncontaminated to moderately contaminated
1 < I_geo_ ≤ 2	2	Moderately contaminated
2 < I_geo_ ≤ 3	3	Moderately to heavily contaminated
3 < I_geo_ ≤ 4	4	Heavily contaminated
4 < I_geo_ ≤ 5	5	Heavily to extremely contaminated
I_geo_ > 5	6	Extremely contaminated

* Wei et al. [17], Aiman et al. [54], Ali et al. [36].

**Table 2:** Values of exposure factors for heavy metals doses for children and adults.

Factor	Description	Unit	Value		References
			Children	Adults	
C	Concentration of metals in dusts	mg/kg			Present study
IngR	Ingestion rate of dust	mg/day	200	100	ESAG [67]; USEPA [65,66]
EF	Exposure frequency	days/year	350	350	Peng et al. [68]; Zheng et al. [18]; ESAG [67]
ED	Exposure duration	years	6	24	USEPA [69]; USEPA [65,66]
BW	Average body weight	kg	15	70	Lappalainen and Knuuttila [70]; Lu et al. [71]; Zheng et al. [18], ESAG [67], USEPA [72]
AT	Average time	days	365 × ED	365 × ED	USEPA [72]
CF	Conversion factor	kg/mg	1 × 10^−6^	1 × 10^−6^	Li et al. [73]
InhR	Inhalation rate of dust	m^3^/day	7.63	12.8	Li et al. [73,74]; USEPA [69]
PEF	Particular emission factor	m^3^/kg	1.36 × 10^9^	1.36 × 10^9^	USEPA [65,66]
SA	Surface area of skin exposed to dust	cm^2^	1600	4350	Zheng et al. [18]; ESAG [67]
AF	Skin adherence factor	mg/cm^2^	0.2	0.7	USEPA [75]; Man et al. [76]
ABF	Absorption factor (Dermal)	unitless	0.001	0.001	Wei et al. [17]; USEPA [65,66]; US Department of Energy [77]

**Table 3:** Concentrations (mg/kg) of heavy metals in road dusts of different functional areas.

Sites		Heavy Metals										
		Fe	Mn	Zn	Pb	Cd	V	Co	Ni	As	Cr	Cu
RA	Min	12,259.00	416.00	304.72	85.00	4.67	51.00	7.63	32.00	13.30	42.44	86.88
	Max	14,769.00	565.00	398.00	115.00	6.25	68.00	9.90	43.30	18.63	55.00	124.00
	Mean	13,543.06	496.03	346.43	100.03	5.31	59.60	8.67	38.20	15.70	48.22	100.69
	SD	996.92	48.88	31.62	10.08	0.53	5.79	0.85	4.06	1.84	4.50	12.32
SA	Min	11,544.72	453.00	395.01	104.00	5.30	63.00	8.60	40.50	16.00	50.30	107.00
	Max	14,693.28	579.00	516.00	148.00	7.80	87.00	13.30	56.20	24.00	70.00	149.00
	Mean	13,119.00	513.01	448.94	129.67	6.90	74.80	10.89	47.91	20.05	59.90	127.70
	SD	1133.61	45.65	42.02	14.34	0.87	8.10	1.47	5.65	2.82	7.07	14.23
MCRA	Min	10,272.00	450.00	398.00	110.00	5.70	65.00	9.20	41.00	17.00	51.00	110.00
	Max	13,528.00	598.00	550.00	157.00	8.50	93.00	13.00	60.00	25.00	74.00	155.50
	Mean	11,900.00	525.00	472.50	136.01	7.20	78.81	11.41	50.04	21.00	63.01	134.39
	SD	1122.98	51.33	52.65	15.72	0.91	9.46	1.39	6.54	2.55	7.58	15.14
PA	Min	9850.00	482.70	442.70	120.00	6.50	67.00	9.50	45.00	19.00	57.80	120.00
	Max	12,800.00	647.00	617.10	180.00	9.50	100.00	14.40	66.00	28.00	83.00	178.00
	Mean	11,200.14	570.01	534.60	154.41	8.20	87.50	12.50	55.00	23.31	71.30	152.11
	SD	1091.17	57.45	59.05	20.57	1.04	11.38	1.62	7.07	2.92	8.35	19.49
TA	Min	10,691.00	550.00	525.00	142.00	7.60	81.50	11.50	51.00	24.38	70.00	140.00
	Max	14,180.00	740.00	736.00	213.00	11.10	125.00	18.00	77.00	31.03	101.00	211.00
	Mean	12,485.03	649.01	635.11	183.52	9.71	103.92	14.80	65.30	27.70	84.72	180.67
	SD	1210.61	65.48	72.47	23.89	1.22	13.75	2.03	8.61	2.39	10.55	23.10
RUA	Min	17,080.00	466.40	71.28	12.70	0.35	30.00	4.05	19.36	1.98	35.50	16.80
	Max	19,920.00	572.40	90.72	17.00	0.45	39.00	5.15	24.64	2.52	47.00	22.62
	Mean	18,500.00	520.91	81.00	15.21	0.40	34.86	4.61	22.00	2.25	41.04	20.20
	SD	965.13	36.43	7.00	1.42	0.04	3.17	0.41	1.90	0.19	3.74	1.97

Notes: RA, residential area; SA, suburban area; MCRA, mixed commercial residential area; PA, parking area; TR, traffic area; RUA, rural area; Min, minimum; Max, maximum; SD, standard deviation.

**Table 4:** Heavy metals concentrations (mg/kg) in urban road dusts of different cities around the world.

Country	City	Fe	Mn	Zn	Pb	Cd	V	Co	Ni	As	Cr	Cu	Reference
Saudi Arabia	Jeddah	12,449.00	550.61	487.52	140.73	7.46	80.92	11.66	51.29	21.55	65.43	139.11	Present study
China	Chengdu	NA	NA	296	82.5	1.66	NA	NA	24.5	NA	84.3	100	Li et al. [35]
Colombia	Villavicencio	NA	NA	210	467	NA	NA	NA	22.3	NA	26	213	Trujillo-Gonzalez et al. [87]
China	Beijing	NA	NA	222	105	0.72	NA	NA	25.2	NA	84.7	69.9	Wei et al. [17]
Iran	Isfahan	NA	NA	707	393	2.14	NA	NA	70	NA	82	182	Soltani et al. [2]
China	Guangzhou	NA	NA	1777	388	2.14	NA	NA	41.4	NA	176	192	Huang et al. [91]
Iran	Shiraz	20,254.5	438.5	403.5	115.7	0.5	NA	NA	77.5	6.58	67.2	136.3	Keshavarzi et al. [34]
Iran	Tahran	47,935.7	1215	873.2	257.4	10.7	NA	NA	34.8	NA	33.5	225.3	Saeedi et al. [92]
UK	Newcastle	992	NA	421	NA	1	NA	NA	26	6.4	NA	132	Okorie et al. [93]
Turkey	Tokat	NA	285	63	149	3	NA	NA	65	NA	30	29	Kurt-karakus [94]
China	Nanjing	34,200	646	394	103	1.1	NA	NA	55.9	13.4	126	123	Hu et al. [27]
USA	Massachusetts	NA	NA	240	73	NA	NA	NA	NA	NA	95	105	Apeagyei et al. [12]
Greece	Kavala	NA	NA	272	301	0.2	NA	NA	58	17	196	124	Christonforridis and Stamatis [31]
China	Baoji	NA	NA	715	408	NA	NA	NA	49	NA	NA	123	Lu et al. [95]
Jordan	Amman	NA	NA	358	236	1.7	NA	NA	88	NA	NA	177	Al-khashman [11]
China	Xian	NA	NA	421	231	NA	NA	NA	NA	NA	167	95	Yongming et al. [55]

NA: Not available.

**Table 5:** The enrichment factor (EF) and I_geo_ of heavy metals in road dusts of different functional areas.

Sites		Heavy Metals										
		Fe	Mn	Zn	Pb	Cd	V	Co	Ni	As	Cr	Cu
RA	EF	1.00	2.17	20.56	33.27	110.36	1.84	1.44	2.12	36.33	2.00	7.61
	I_geo_	−2.64	−1.52	1.72	2.42	4.15	−1.76	−2.11	−1.56	2.54	−1.64	0.29
SA	EF	1.00	2.32	27.52	44.52	147.69	2.38	1.87	2.74	47.56	2.57	9.96
	I_geo_	−2.69	−1.47	2.10	2.79	4.52	−1.44	−1.79	−1.23	2.89	−1.33	0.63
MCRA	EF	1.00	2.61	31.93	51.66	171.38	2.76	2.17	3.18	55.20	2.98	11.56
	I_geo_	−2.83	−1.44	2.17	2.86	4.59	−1.36	−1.71	−1.16	2.96	−1.25	0.70
PA	EF	1.00	3.02	38.39	62.11	206.03	3.26	2.51	3.68	65.12	3.58	13.90
	I_geo_	−2.91	−1.32	2.35	3.04	4.77	−1.21	−1.59	−1.03	3.11	−1.07	0.88
TA	EF	1.00	3.08	40.91	66.19	219.57	3.47	2.67	3.93	69.41	3.82	14.81
	I_geo_	−2.76	−1.13	2.60	3.29	5.02	−0.96	−1.34	−0.79	3.36	−0.82	1.13
Mean (Urban dust)	EF	1.00	2.62	31.50	50.91	168.78	2.71	2.11	3.09	54.15	2.96	11.44
	I_geo_	−2.76	−1.37	2.22	2.91	4.64	−1.32	−1.69	−1.13	3.00	−1.20	0.75
RUA	EF	1.00	1.67	3.52	3.70	6.09	0.79	0.56	0.89	3.80	1.25	1.12
	I_geo_	−2.19	−1.45	−0.37	−0.30	0.42	−2.54	−3.03	−2.35	−0.26	−1.87	−2.03

Notes: RA, residential area; SA, suburban area; MCRA, mixed commercial residential area; PA, Parking area; TR, traffic area; RUA, rural area.

**Table 6:** Hazard quotient and hazard index of each heavy metal for children population living in different functional areas.

Risk	Area	Heavy Metals										
		Fe	Mn	Zn	Pb	Cd	V	Co.	Ni	As	Cr	Cu
HQ_ing_	RA	2.06E−02	1.35E−01	1.48E−02	3.65E−01	6.79E−02	1.09E−01	5.54E−03	2.44E−02	6.69E−01	2.06E−01	3.22E−02
	SA	2.00E−02	1.40E−01	1.91E−02	4.74E−01	8.82E−02	1.37E−01	6.96E−03	3.06E−02	8.54E−01	2.55E−01	4.08E−02
	MCRA	1.81E−02	1.43E−01	2.01E−02	4.97E−01	9.21E−02	1.44E−01	7.29E−03	3.20E−02	8.95E−01	2.69E−01	4.30E−02
	PA	1.70E−02	1.55E−01	2.28E−02	5.64E−01	1.05E−01	1.60E−01	7.99E−03	3.52E−02	9.93E−01	3.04E−01	4.86E−02
	TA	1.90E−02	1.77E−01	2.71E−02	6.70E−01	1.24E−01	1.90E−01	9.46E−03	4.17E−02	1.18E+00	3.61E−01	5.77E−02
	RUA	2.82E−02	1.42E−01	3.45E−03	5.56E−02	5.11E−03	6.37E−02	2.95E−03	1.41E−02	9.59E−02	1.75E−01	6.46E−03
HQ_inh_	RA	2.21E−02	1.24E−02	4.14E−07	1.02E−05	1.90E−06	3.05E−06	5.45E−04	6.65E−07	1.87E−05	6.05E−04	8.98E−07
	SA	2.14E−02	1.29E−02	5.37E−07	1.32E−05	2.47E−06	3.83E−06	6.84E−04	8.34E−07	2.39E−05	7.51E−04	1.14E−06
	MCRA	1.94E−02	1.32E−02	5.65E−07	1.39E−05	2.58E−06	4.04E−06	7.17E−04	8.71E−07	2.50E−05	7.90E−04	1.20E−06
	PA	1.83E−02	1.43E−02	6.39E−07	1.57E−05	2.94E−06	4.48E−06	7.85E−04	9.58E−07	2.78E−05	8.94E−04	1.36E−06
	TA	2.04E−02	1.63E−02	7.59E−07	1.87E−05	3.48E−06	5.32E−06	9.30E−04	1.14E−06	3.30E−05	1.06E−03	1.61E−06
	RUA	3.02E−02	1.31E−02	9.68E−08	1.55E−06	1.43E−07	1.79E−06	2.90E−04	3.83E−07	2.68E−06	5.15E−04	1.80E−07
HQ_derm_	RA	3.96E−03	5.51E−03	1.18E−04	3.90E−03	1.09E−02	1.74E−02	1.11E−05	1.45E−04	2.61E−03	1.97E−02	1.72E−04
	SA	3.83E−03	5.70E−03	1.53E−04	5.05E−03	1.41E−02	2.19E−02	1.39E−05	1.81E−04	3.33E−03	2.45E−02	2.18E−04
	MCR	3.48E−03	5.84E−03	1.61E−04	5.30E−03	1.47E−02	2.30E−02	1.46E−05	1.90E−04	3.46E−03	2.58E−02	2.29E−04
	PA	3.27E−03	6.34E−03	1.82E−04	6.02E−03	1.68E−02	2.56E−02	1.60E−05	2.08E−04	3.88E−03	2.92E−02	2.59E−04
	TA	3.65E−03	7.22E−03	2.17E−04	7.15E−03	1.99E−02	3.04E−02	1.89E−05	2.47E−04	4.61E−03	3.47E−02	3.08E−04
	RUA	5.41E−03	5.79E−03	2.76E−05	5.93E−04	8.18E−04	1.02E−02	5.89E−06	8.33E−05	3.74E−04	1.68E−02	3.44E−05
HI	RA	4.66E−02	1.53E−01	1.49E−02	3.69E−01	7.88E−02	1.26E−01	6.10E−03	2.46E−02	6.72E−01	2.26E−01	3.24E−02
	SA	4.52E−02	1.58E−01	1.93E−02	4.79E−01	1.02E−01	1.58E−01	7.66E−03	3.08E−02	8.58E−01	2.81E−01	4.10E−02
	MCRA	4.10E−02	1.62E−01	2.03E−02	5.02E−01	1.07E−01	1.67E−01	8.03E−03	3.22E−02	8.98E−01	2.95E−01	4.32E−02
	PA	3.86E−02	1.76E−01	2.30E−02	5.70E−01	1.22E−01	1.85E−01	8.79E−03	3.54E−02	9.97E−01	3.34E−01	4.89E−02
	TA	4.30E−02	2.00E−01	2.73E−02	6.78E−01	1.44E−01	2.20E−01	1.04E−02	4.20E−02	1.19E+00	3.97E−01	5.81E−02
	RUA	6.37E−02	1.61E−01	3.48E−03	5.62E−02	5.93E−03	7.39E−02	3.24E−03	1.41E−02	9.63E−02	1.92E−01	6.49E−03
R_f_D_ing_		8.40E+00	4.70E−02	3.00E−01	3.50E−03	1.00E−03	7.00E−03	2.00E−02	2.00E−02	3.00E−04	3.00E−03	4.00E−02
R_f_D_inh_		2.20E−04	1.43E−05	3.00E−01	3.52E−03	1.00E−03	7.00E−03	5.71E−06	2.06E−02	3.01E−04	2.86E−05	4.02E−02
R_f_D_derm_		7.00E−02	1.84E−03	6.00E−02	5.25E−04	1.00E−05	7.00E−05	1.60E−02	5.40E−03	1.23E−04	5.00E−05	1.20E−02

**Table 7:** Hazard quotient and hazard index of each heavy metal for adults population living in different functional areas.

Risk	Area	Heavy Metals										
		Fe	Mn	Zn	Pb	Cd	V	Co.	Ni	As	Cr	Cu
HQ_ing_	RA	2.21E−03	1.45E−02	1.58E−03	3.92E−02	7.27E−03	1.17E−02	5.94E−04	2.62E−03	7.17E−02	2.20E−02	3.45E−03
	SA	2.14E−03	1.50E−02	2.05E−03	5.08E−02	9.45E−03	1.46E−02	7.46E−04	3.28E−03	9.16E−02	2.74E−02	4.37E−03
	MCRA	1.94E−03	1.53E−02	2.16E−03	5.32E−02	9.86E−03	1.54E−02	7.82E−04	3.43E−03	9.59E−02	2.88E−02	4.60E−03
	PA	1.83E−03	1.66E−02	2.44E−03	6.04E−02	1.12E−02	1.71E−02	8.56E−04	3.77E−03	1.06E−01	3.26E−02	5.21E−03
	TA	2.04E−03	1.89E−02	2.90E−03	7.18E−02	1.33E−02	2.03E−02	1.01E−03	4.47E−03	1.26E−01	3.87E−02	6.19E−03
	RUA	3.02E−03	1.52E−02	3.70E−04	5.95E−03	5.48E−04	6.82E−03	3.16E−04	1.51E−03	1.03E−02	1.87E−02	6.92E−04
HQ_inh_	RA	7.94E−03	4.47E−03	1.49E−07	3.66E−06	6.85E−07	1.10E−06	1.96E−04	2.39E−07	6.72E−06	2.17E−04	3.23E−07
	SA	7.69E−03	4.63E−03	1.93E−07	4.75E−06	8.90E−07	1.38E−06	2.46E−04	3.00E−07	8.59E−06	2.70E−04	4.10E−07
	MCRA	6.97E−03	4.73E−03	2.03E−07	4.98E−06	9.28E−07	1.45E−06	2.58E−04	3.13E−07	8.99E−06	2.84E−04	4.31E−07
	PA	6.56E−03	5.14E−03	2.30E−07	5.66E−06	1.06E−06	1.61E−06	2.82E−04	3.44E−07	9.98E−06	3.21E−04	4.88E−07
	TA	7.32E−03	5.85E−03	2.73E−07	6.72E−06	1.25E−06	1.91E−06	3.34E−04	4.09E−07	1.19E−05	3.82E−04	5.79E−07
	RUA	1.08E−02	4.70E−03	3.48E−08	5.57E−07	5.16E−08	6.42E−07	1.04E−04	1.38E−07	9.64E−07	1.85E−04	6.48E−08
HQ_derm_	RA	8.07E−03	1.12E−02	2.41E−04	7.95E−03	2.21E−02	3.55E−02	2.26E−05	2.95E−04	5.32E−03	4.02E−02	3.50E−04
	SA	7.82E−03	1.16E−02	3.12E−04	1.03E−02	2.88E−02	4.46E−02	2.84E−05	3.70E−04	6.80E−03	5.00E−02	4.44E−04
	MCRA	7.09E−03	1.19E−02	3.28E−04	1.08E−02	3.00E−02	4.70E−02	2.97E−05	3.87E−04	7.12E−03	5.26E−02	4.67E−04
	PA	6.67E−03	1.29E−02	3.72E−04	1.23E−02	3.42E−02	5.21E−02	3.26E−05	4.25E−04	7.90E−03	5.95E−02	5.29E−04
	TA	7.44E−03	1.47E−02	4.42E−04	1.46E−02	4.05E−02	6.19E−02	3.86E−05	5.04E−04	9.39E−03	7.07E−02	6.28E−04
	RUA	1.10E−02	1.18E−02	5.63E−05	1.21E−03	1.67E−03	2.08E−02	1.20E−05	1.70E−04	7.63E−04	3.42E−02	7.02E−05
HI	RA	1.82E−02	3.02E−02	1.82E−03	4.71E−02	2.94E−02	4.72E−02	8.12E−04	2.91E−03	7.70E−02	6.25E−02	3.80E−03
	SA	1.76E−02	3.12E−02	2.36E−03	6.11E−02	3.82E−02	5.92E−02	1.02E−03	3.65E−03	9.84E−02	7.76E−02	4.82E−03
	MCRA	1.60E−02	3.19E−02	2.49E−03	6.40E−02	3.99E−02	6.24E−02	1.07E−03	3.81E−03	1.03E−01	8.16E−02	5.07E−03
	PA	1.51E−02	3.47E−02	2.81E−03	7.27E−02	4.54E−02	6.93E−02	1.17E−03	4.19E−03	1.14E−01	9.24E−02	5.74E−03
	TA	1.68E−02	3.95E−02	3.34E−03	8.64E−02	5.38E−02	8.23E−02	1.39E−03	4.98E−03	1.36E−01	1.10E−01	6.82E−03
	RUA	2.49E−02	3.17E−02	4.26E−04	7.16E−03	2.22E−03	2.76E−02	4.32E−04	1.68E−03	1.10E−02	5.32E−02	7.62E−04
R_f_D_ing_		8.40E+00	4.70E−02	3.00E−01	3.50E−03	1.00E−03	7.00E−03	2.00E−02	2.00E−02	3.00E−04	3.00E−03	4.00E−02
R_f_D_inh_		2.20E−04	1.43E−05	3.00E−01	3.52E−03	1.00E−03	7.00E−03	5.71E−06	2.06E−02	3.01E−04	2.86E−05	4.02E−02
R_f_D_derm_		7.00E−02	1.84E−03	6.00E−02	5.25E−04	1.00E−05	7.00E−05	1.60E−02	5.40E−03	1.23E−04	5.00E−05	1.20E−02

**Table 8:** Hazard quotient, hazard index and carcinogenic risk of average concentrations of each heavy metals for both children and adults population living in urban areas of Jeddah.

Risk		Heavy Metals										
		Fe	Mn	Zn	Pb	Cd	V	Co.	Ni	As	Cr	Cu
HQ_ing_	Children	1.89E−02	1.50E−01	2.08E−02	5.14E−01	9.54E−02	1.48E−01	7.45E−03	3.28E−02	9.18E−01	2.79E−01	4.45E−02
	Adults	2.03E−03	1.60E−02	2.23E−03	5.51E−02	1.02E−02	1.58E−02	7.99E−04	3.51E−03	9.84E−02	2.99E−02	4.76E−03
HQ_inh_	Children	2.03E−02	1.38E−02	5.83E−07	1.43E−05	2.68E−06	4.15E−06	7.32E−04	8.93E−07	2.57E−05	8.21E−04	1.24E−06
	Adults	7.30E−03	4.96E−03	2.10E−07	5.15E−06	9.62E−07	1.49E−06	2.63E−04	3.21E−07	9.23E−06	2.95E−04	4.46E−07
HQ_derm_	Children	3.64E−03	6.12E−03	1.66E−04	5.48E−03	1.53E−02	2.36E−02	1.49E−05	1.94E−04	3.58E−03	2.68E−02	2.37E−04
	Adults	7.42E−03	1.25E−02	3.39E−04	1.12E−02	3.11E−02	4.82E−02	3.04E−05	3.96E−04	7.31E−03	5.46E−02	4.84E−04
HI	Children	4.29E−02	1.70E−01	2.09E−02	5.20E−01	1.11E−01	1.71E−01	8.20E−03	3.30E−02	9.22E−01	3.06E−01	4.47E−02
	Adults	1.67E−02	3.35E−02	2.57E−03	6.63E−02	4.13E−02	6.41E−02	1.09E−03	3.91E−03	1.06E−01	8.48E−02	5.25E−03
CRA	Children				1.12E−08	4.42E−07		1.07E−06	4.05E−07	3.06E−08	2.58E−07	
	Adults				1.20E−09	4.73E−08		1.15E−07	4.34E−08	3.28E−09	2.77E−08	

**Table 9:** Carcinogenic risk (CRA) of each heavy metal for children and adults population living in different functional areas.

Area		Heavy Metals					
		Pb	Cd	Co	Ni	As	Cr
RA	Children	7.99E−09	3.14E−07	7.99E−07	3.02E−07	2.23E−08	1.90E−07
	Adults	8.56E−10	3.37E−08	8.56E−08	3.23E−08	2.39E−09	2.04E−08
SA	Children	1.04E−08	4.09E−07	1.00E−06	3.78E−07	2.85E−08	2.37E−07
	Adults	1.11E−09	4.38E−08	1.07E−07	4.05E−08	3.05E−09	2.53E−08
MCRA	Children	1.09E−08	4.26E−07	1.05E−06	3.95E−07	2.98E−08	2.49E−07
	Adults	1.16E−09	4.57E−08	1.13E−07	4.23E−08	3.19E−09	2.67E−08
PA	Children	1.23E−08	4.86E−07	1.15E−06	4.34E−07	3.31E−08	2.82E−07
	Adults	1.32E−09	5.20E−08	1.23E−07	4.65E−08	3.55E−09	3.02E−08
TA	Children	1.47E−08	5.75E−07	1.36E−06	5.16E−07	3.93E−08	3.35E−07
	Adults	1.57E−09	6.16E−08	1.46E−07	5.52E−08	4.21E−09	3.58E−08
RUA	Children	1.22E−09	2.37E−08	4.25E−07	1.74E−07	3.19E−09	1.62E−07
	Adults	1.30E−10	2.54E−09	4.55E−08	1.86E−08	3.42E−10	1.74E−08
Sf_inh_		8.50E−03	6.30E+00	9.80E+00	8.40E−01	1.51E−01	4.20E−01

## Supplementary Materials

The following are available online at www.mdpi.com/1660-4601/15/1/36/s1, Table S1: Certified and measured values and recovery [C(element measured)/ C(element certified) × 100,%] of each tested element in certified reference material for the present study.
