# Supplementary material for: Risk Assessment and Implication of Human Exposure to Road Dust Heavy Metals in Jeddah, Saudi Arabia
Source: Int J Environ Res Public Health. 2017 Dec 26;15(1):36. doi: 10.3390/ijerph15010036 (PMC5799873; doi:10.3390/ijerph15010036)
Supplement: Supplementary file 1 [file ijerph-15-00036-s001.pdf]

Supplementary Materials: Risk Assessment and Implication of Human Exposure to Road Dust Heavy Metals in Jeddah, Saudi Arabia

Ibrahim I. Shabbaj, Mansour A. Alghamdi *, Magdy Shamy, Salwa K. Hassan, Musaab M. Alsharif and Mamdouh I. Khoder

Table S1. Certified and measured values and recovery [C(element, measured)/C(element, certified) × 100, %] of each tested element in certified reference material for the present study.

Elements	NIST 2710		
	Certified Values (mg/kg)	Measured Values (mg/kg)	Recovery (%)
Mn	1.01	0.97	95.60
As	626.00	599.08	95.70
Fe	3.38	3.06	90.50
Cd	21.80	19.97	91.60
Co	10.00	8.27	82.70
Cr	39.00	28.55	73.20
Cu	2950.00	2303.95	78.10
Ni	14.30	14.10	98.60
Pb	5532.00	5692.43	102.90
Zn	6952.00	6896.38	99.20
V	76.60	72.00	94.00

© 2017 by the authors; licensee MDPI, Basel, Switzerland. This article is an open access article distributed under the terms and conditions of the Creative Commons by Attribution (CC-BY) license (http://creativecommons.org/licenses/by/4.0/).
